# Family dynamics in Filipino transnational families: the role of non-migrant mothers

**DOI:** 10.3389/fsoc.2026.1752009

**Published:** 2026-03-31

**Authors:** Peachy Domingo

**Affiliations:** Department of Social Sciences, College of Arts and Social Sciences, Central Luzon State University, Muñoz, Philippines

**Keywords:** family cohesion, family relations, gender, mediated patriarchy, motherhood, overseas Filipino families, transnational communication, transnational families

## Abstract

Since the 1970s, large-scale Filipino migration has led to the long-term absence and global dispersal of family members, giving rise to Filipino transnational families. This separation affects family stability and interpersonal relationships, yet many families maintain strong bonds across borders. However, the crucial role of non-migrant mothers in sustaining family cohesion often remains overlooked. To address this gap, this descriptive study explores the following: (1) the roles of non-migrant mothers in maintaining family cohesion in overseas Filipino worker (OFW) families and (2) the dynamics of communication and emotional support between Filipino non-migrant mothers, children, and their OFW husbands. Drawing on in-depth qualitative interviews with 20 non-migrant mothers who have OFW husbands in the Philippines, the findings highlight the central position of mothers in maintaining a sense of family hood. The study shows that non-migrant mothers act as moral gatekeepers, embodying ideals of intensive motherhood within traditional Filipino family values to avoid social scrutiny and adhere to expectations of maternal responsibility. In addition to caregiving, they also serve as mediators in the father–child relationship, facilitating their husbands’ symbolic presence and enabling migrant fathers to remain influential figures in their children’s lives. However, this mediating role simultaneously reproduces mediated patriarchy. By sustaining the father’s authority from afar, non-migrant mothers enable forms of surveillance over daily family life and financial management that extend beyond migrant fathers’ direct involvement in co-parenting. At the same time, mothers frequently assume co-provider roles, although their capacity to do so varies—from survival-level contributions to significant economic participation—reflecting differences in socioeconomic positioning. How mothers make sense of their labor force participation and financial contributions, in turn, shapes their perceived authority within the family vis-à-vis their OFW husbands. The study further underscores the contradictory role of transnational communication. On the one hand, it is essential for emotional connection and interactional adjustment, which helps preserve a sense of family hood across distance. Positioned at the center of OFW families, non-migrant mothers function as key conduits between migrant fathers and children. On the other hand, particularly in households where mothers are full-time caregivers, transnational communication can reinforce mediated patriarchy by sustaining paternal oversight. Grounded in family systems theory, this study highlights the indispensable yet ambivalent role of non-migrant mothers in maintaining family cohesion within Filipino transnational families.

## 1 Introduction

Approximately 2.16 million Overseas Filipino Workers (OFWs) were employed abroad between April and September 2023, according to the Philippine Statistics Authority (2023). By the last quarter of 2023, at least 6.3% of Filipino households had members who were either currently or previously employed as OFWs (Balita, 2024a). More than half are married and have children at the time of migration (Parreño, 2022). Poverty has consistently been identified as a major driver of labor migration, compelling many Filipinos to seek employment opportunities overseas (Gozum, 2020). At the macro level, the deployment of OFWs serves as one of the most effective strategies for poverty alleviation in the absence of substantial foreign direct investments (Opiniano, 2024). Asis (1994) also highlights how overseas labor migration has become a powerful driver of social mobility for ordinary Filipino families.

The large-scale migration of Filipino workers began in the 1970s when the Philippine government facilitated the deployment of male contract workers to the Middle East. These workers were primarily engaged in infrastructure projects in the oil-rich Gulf countries, which were experiencing rapid development during that period (Asis, 2004). Since then, overseas labor migration has become deeply embedded in the Philippine economy. In 2023 alone, OFWs contributed a total of 33.5 billion US dollars in remittances (Balita, 2024b). The long-term nature of overseas employment is reflected in the findings of a 2016 survey by the Department of Labor and Employment - Institute of Labor Studies, which reported that male OFWs spent up to 34 years working abroad (Parreño, 2022), underscoring the sustained reliance of migrant-sending households on overseas labor.

The prolonged absence and dispersal of family members across national borders due to overseas work (Saksela-Bergholm, 2019) has led to the emergence of transnational families. This transformation in family structure reshapes everyday family life, affecting the dynamics, resilience, and potential fragility of familial bonds (Gotehus, 2023). In the Philippine context, such separation raises concerns regarding family stability, particularly in terms of marital relationships and family cohesion (Asis, 2013). Earlier studies similarly warned that increasing Filipino overseas labor migration could disrupt family role systems, potentially resulting in role overload among non-migrant spouses and straining relationships within the family system (Medina and De Guzman, 1994).

Despite the challenges posed by physical separation, transnational families continue to sustain strong family ties and kinship networks that extend across national borders, maintaining a sense of “familyhood” (Christou and Kofman, 2022). Central to transnational familyhood are practices that foster collective belonging, uphold shared responsibilities, and sustain caring relationships and family expectations across distances (Christou and Kofman, 2022).

Within these arrangements, the traditional gendered division of labor remains central to transnational family organizations. However, the COVID-19 pandemic significantly disrupted temporary labor migration, exposing male OFWs to heightened labor precarity (Banta and Pratt, 2023). Filipino maritime workers, for instance, were quarantined without pay, while others were forcibly repatriated due to terminated contracts, border closures, and limited opportunities to return to overseas employment (Francisco-Menchavez, 2024). Consequently, many OFWs were left without wages, highlighting the increased vulnerability of transnational families during the pandemic. OFWs who experienced involuntary returns were compelled to draw prematurely on resources intended for the future, depleting savings or liquidating assets to meet daily expenses (Liao, 2025). These disruptions not only intensified the economic vulnerability of transnational families but also shifted the burden of maintaining household stability and family cohesion onto those who remained behind—particularly non-migrant mothers.

While the majority of the existing studies has focused on how Filipino migrant mothers maintain relationships with their children and non-migrant husbands (Garabiles, 2020), less attention has been given to the experiences of non-migrant mothers in OFW families. Despite being understudied, their role in sustaining male emigration is critical. First, they assume the responsibilities and obligations left behind by their emigrant husbands (Galam, 2015), often reshaping their roles within the family. Second, they play a pivotal role in maintaining family cohesion, establishing social arrangements, and ensuring the financial wellbeing of their households, all while navigating the emotional and logistical challenges of transnational life (Kanaiaupuni, 2000).

Previous studies have underscored the central role of non-migrant mothers in sustaining transnational family life. Carling et al. (2012) highlighted how non-migrant mothers continuously provide care work for their children in the absence of migrant fathers. Similarly, Pinzon (2021) found that Filipino non-migrant mothers often act as intermediaries between their OFW husbands and their children. In doing so, they may encourage children to withhold unfavorable news from their fathers to protect them from worry and stress. While this mediating practice is intended to maintain emotional stability, it can inadvertently create emotional distance, as children suppress their personal concerns within this managed relationship.

Maintaining stability in transnational families thus relies heavily on the efforts of non-migrant mothers. However, their economic contributions are often overshadowed by the economic role of migrant husbands (Gartaula et al., 2012). Highlighting the experiences of non-migrant wives is therefore essential to understanding how migration reshapes gendered roles and responsibilities within families (Yabiku et al., 2010). In the Philippines, Filipino culture dictates that women are responsible for maintaining family stability and providing a caring and nurturing relationship with their families, even amid overseas labor migration. Given this context, this study unravels the experiences of non-migrant mothers from OFW families.

The purpose of this study is to describe the roles and responsibilities of non-migrant mothers in OFW families and examine their impact on family cohesion. In addition, it seeks to explore the dynamics of communication and emotional support between Filipino non-migrant mothers, their children, and their OFW husbands.

## 2 Literature review

A review of previous studies on motherhood is presented to examine how non-migrant mothers construct their understanding of motherhood. The literature highlights that motherhood is deeply rooted in cultural, societal, and gender norms.

### 2.1 Motherhood in the Philippines

The prevailing notion of a “good mother” traditionally implies that women are the primary caregivers, best suited for nurturing and looking after their children (Williamson et al., 2023). This expectation often positions mothers as the central figures responsible for their children’s wellbeing, shaping both societal attitudes and personal choices regarding motherhood.

While the notion of a *“good mother”* varies across cultures, certain responsibilities remain consistent. For example, in the Filipino context, mothers are seen as nurturing caregivers who must ensure that their families’ basic needs, such as food and financial stability, are met (Jabar, 2020). Despite the shifting roles, Filipino mothers continue to be held accountable for their children’s wellbeing, reflecting the enduring cultural and societal expectations surrounding motherhood.

Beyond caregiving, mothers are often viewed as moral gatekeepers, tasked with raising morally upright and socially responsible citizens (Liamputtong, 2006). This perspective frames motherhood as not only a practical but also a moral obligation, placing immense pressure on women to ensure their children’s ethical development (Valencia, 2015).

For working Filipino mothers, balancing financial contributions with nurturing duties creates a complex dynamic. Despite entering the workforce, many Filipino women still shoulder the bulk of household and caregiving tasks, a phenomenon described by Hochschild and Machung (1989) as the “second shift.” These dual responsibilities often cause working mothers to leave the workforce in favor of childrearing, which is framed as a maternal preference, although it frequently results from societal pressure to prioritize motherhood (Sakaluk et al., 2022).

### 2.2 Mothering practices

Generally, mothering practices reflect societal norms and gender expectations, including reproductive tasks that shape women’s identities (Delgado-Herrera et al., 2024). However, specific historical contexts—such as periods of overseas labor migration, globalization, and shifts in traditional family roles and structures—also play a significant role in shaping these practices (Wahyuni, 2005). For example, in Indonesia, Wahyuni (2005) found that migration led to the division of nuclear families into separate households, disrupting traditional family roles and reshaping family dynamics. Similarly, migration has affected the composition of Filipino households (Palac, 2005). In transnational Filipino families where mothers are away, Parrenas (2005) found that despite the reorganization of households, gender boundaries persist. Fathers reduce their involvement in housework, while female relatives take on household responsibilities, and migrant mothers continue to nurture from a distance, thereby reinforcing traditional domestic duties.

This pattern aligns with global theories on collectivist cultures, where maternal practices emphasize interdependence, respect for elders, and deference to authority (Alampay and Jocson, 2011). These expectations hold true irrespective of whether Filipino mothers are employed or full-time caregivers.

Previous studies have shown that the parenting style of mothers impacts the parent–child relationship (Bojczyk et al., 2011), particularly in the interplay between child autonomy and maternal authority (Kapetanovic et al., 2019; Valizadeh et al., 2018). Thus, Kuppens and Ceulemans (2019) suggested that to strengthen the relationship and promote family cohesion, parental support should outweigh control in parent–child relationships. This context should be considered in OFW families. However, the focus of research on OFW families is primarily on families with transnational mothers and non-migrant fathers (Garabiles, 2020; Parrenas, 2005). There is a scarcity of studies focusing on the role of non-migrant wives in maintaining family solidarity within parent–child and marital relationships.

### 2.3 Motherhood in transnational families

Filipinos are deeply family-oriented, with close family ties forming the core of their identity. Grounded in Asia’s collectivist culture, this strong sense of connection emphasizes kinship and unity, often placing family and collective wellbeing above individual pursuits. According to Morillo et al. (2013), the Filipino family system is characterized by reciprocity, prioritizing internal relationships and group dynamics, where individual experiences are both shaped by and shape familial and societal influences.

Motherhood in Filipino transnational families is influenced by socio-cultural factors, particularly the gendered division of labor. According to Parrenas (2001), fathers are expected to be the primary breadwinners, while mothers take on the responsibility of maintaining family life. However, compared to other Asian countries, the Philippines has a more egalitarian gender structure, which enables mothers to work overseas to support their children.

Research in the Philippines on transnational families examines family dynamics and gender negotiations between overseas migrant wives and their non-migrant husbands (Arguillas et al., 2018; Asis, 2004; Garabiles, 2020). Other studies focus on how transnational families are maintained in mother-away families (Peng and Wong, 2016). Parrenas (2005) studied the gender paradox in OFW families and concluded that in mother-migrant families, the social reproduction of the conventional notion of a mother’s domestic responsibilities maintains family cohesion. Furthermore, socio-cultural norms in the Philippines pressure OFW families to uphold traditional gender norms and cultural expectations. Similarly, in Vietnamese transnational families, maternal overseas labor migration does not displace gendered moral obligations, as migrant mothers continue to be held primarily responsible for their children’s emotional wellbeing (Hoang and Yeoh, 2011). In contrast, among Indonesian women in transnational families—specifically Indonesian Australian families in Canberra—motherhood is experienced through active engagement in struggles over the recognition of their children’s dual citizenship (Winarnita, 2008). These struggles transform maternal roles into forms of political motherhood, revealing how citizenship regimes constitute a site of contestation for mothers in transnational biracial families.

### 2.4 Family systems theory

The family systems theory provides a framework for examining the roles of non-migrant mothers within the context of overseas migration among OFWs. This theory highlights how the family operates as a dynamic system composed of interconnected subsystems, such as parent–child, marital dyad, and triad relationships. Family functioning is shaped by both socio-cultural factors and interpersonal dynamics among its members. As noted by Fingerman and Berman (2000), physical separation—such as that caused by migration—significantly impacts how family members interact and relate to one another. In OFW families, it is common for one or both parents—often the mother or father—to work abroad, leaving children in the care of the remaining parent. This separation alters family dynamics and role relationships. In mother-away migrant families, non-migrant fathers often take on the role of primary caregiver, sometimes becoming househusbands. Conversely, in father-away migrant families, non-migrant mothers frequently experience role overload, as they manage both caregiving and breadwinning responsibilities. These shifts inevitably affect the balance and functioning of the family unit.

This study aims to provide a sociological analysis of the roles of non-migrant mothers in OFW families. It focuses on their lived experiences and the challenges they face in maintaining family cohesion and fostering strong relationships between their OFW husbands and children. Anchored in family systems theory and informed by the socio-cultural construction of motherhood in Filipino society, this paper explores how both individual and cultural factors influence the functioning of non-migrant mothers in these transnational family arrangements.

## 3 Methodology

Grounded in descriptive phenomenological research, the methodology aimed to describe the role of non-migrant mothers in relation to family dynamics during paternal overseas employment. This research explores the experiences of non-migrant mothers and how the overseas migration of their OFW husbands shapes their roles. It examines how fulfilling these roles promotes family cohesion and explores the significant role of context in shaping the mothers’ identities (Creswell and Poth, 2018).

Using a qualitative framework with in-depth interviews, data was collected. The province of Nueva Ecija was chosen as the area of study. According to the Philippine Statistics Authority, there were around 1.6 million overseas Filipino workers in 2022. Nueva Ecija is in Central Luzon, the second-largest region contributing the highest number of OFWs, accounting for 13.3% of the total OFW population in the Philippines (Philippine Statistics Authority, 2023).

The informants were selected through purposive sampling with the following criteria: legally married to an OFW and a mother to an adolescent child aged 10–19 years. A substantial amount of research claims that the transition to adolescence encompasses several shifts in the mother-child relationship marked by higher odds of emotional detachment and distance, decreased intimate interaction resulting from heightened demand for adolescents’ sense of individuation, and more significant association and intimacy with friends (Larson and Richards, 1991). Anonymity and confidentiality were ensured using pseudonyms; to reduce bias, adolescents were not given access to their mothers’ reports.

Informants exhibit heterogeneity regarding the remittances they receive from their OFW husbands. While some find it necessary to seek additional sources of income, others perceive the remittances as sufficient, thereby foregoing the need to contribute financially to their spouses. Additionally, the informants display diversity in terms of educational attainment, representing a spectrum of educational levels.

A total of 20 informants were interviewed, at which point theoretical saturation was achieved. Due to pandemic restrictions, online interviews were recorded with informants’ consent. After completing individual interviews, the recordings were transcribed.

Confidentiality and anonymity are upheld in this study. Informants were informed that they could withdraw from the interview at any time without any negative consequences. Colleagues with expertise in psychology were available for referrals if informants experienced emotional distress during the interview. However, the interview atmosphere was generally light-hearted, with some informants laughing while discussing their children. One participant, who had recently experienced her husband leaving for another family, displayed a more somber tone. Overall, the informants reported that they enjoyed the interview process, which resembled a conversation between two mothers. As the interviews concluded, not only did the researcher express gratitude to the informants, but they also thanked the researcher for providing them with an opportunity to share their experiences in mothering adolescent children.

The data were analyzed using thematic analysis, following the six-phase framework proposed by Braun and Clarke (2021): familiarization with the data, generation of initial codes, searching for themes, reviewing potential themes, defining and naming themes, and writing up the findings (Ahmed et al., 2025; Langdridge, 2007). The familiarization phase involved immersing oneself in the transcriptions (Cohen et al., 2000). Transcripts were reviewed repeatedly to familiarize oneself with the data and construct a narrative for each informant. Subsequently, an individual data matrix was developed for each informant to facilitate the process before proceeding to open coding. A consolidated matrix encompassing all informants was then created to enable cross-case analysis. Recurring and salient patterns across informants’ accounts served as the basis for theme development, particularly in relation to the role of non-migrant mothers in promoting family cohesion and the dynamics of communication and support within transnational families.

## 4 Findings

The major themes derived from the data are: (1) the role of non-migrant mothers in sustaining family cohesion in OFW households, and (2) communication dynamics and emotional support in OFW families. These themes reflect the experiences and narratives of non-migrant mothers from OFW families.

### 4.1 The role of non-migrant mothers in sustaining family cohesion in OFW households

The migration of Filipino workers abroad creates significant disruptions in family dynamics, placing the burden of maintaining family cohesion on non-migrant mothers. These women navigate role overload, weakened father–child relationships, and financial instability through adaptive strategies that preserve emotional bonds and economic security. This study highlights their role as primary caregivers, emotional mediators, and financial managers, underscoring the gendered nature of transnational parenting.

#### 4.1.1 Intensive motherhood and the moral burden of parenting

Filipino non-migrant mothers largely embrace traditional maternal roles, assuming primary responsibility for child-rearing, household management, and moral education. This study finds that overseas labor migration reinforces the ideology of intensive motherhood, as 14 out of 20 informants currently fulfill the role of stay-at-home non-migrant wives who strongly believe that mothers are solely accountable for their children’s values formation and household wellbeing. This dynamic sustains the traditional gendered division of labor prevalent in the context of husbands’ overseas employment and the societal expectations placed on non-migrant wives. Notably, this trend persists despite the educational attainment of 12 out of 20 informants, who have completed vocational or college education, and the work experience of eight among them, including four who were previously OFWs (Table 1).

**Table 1:** Profile of non-migrant mothers.

Rank	Mother	Monthly remittance (₱)	Mother’s education	Mother’s employment status	Husband’s education	Years of husband’s migration	Nature of husband’s overseas work	Socio-economic condition
1	Khaye	14,000	High school	Working full-time (house helper)	High school	12	Aluminum company worker (Saudi)	Low—Precarious
2	Myra	15,000	High school	Stay-at-home, previously employed	High school	2	Painter (Qatar)	Low—Precarious
3	Ruby	15,000	Vocational	Stay-at-home, no work experience	Vocational	15	Aluminum company worker (NZ)	Low—Precarious
4	Shannah	15,000	College	Stay-at-home, previously employed (OFW)	College undergraduate	2	Safety officer (Dubai)	Low—Precarious
5	Cherry	16,000–25,000	High school	Working part-time (seasonal farm worker)	College undergraduate	4	Electrician (Saudi)	Low—Precarious
6	Carol	20,000	College	Working full-time (midwife)	College undergraduate	4	Auto mechanic (Saudi)	Lower-Middle—Vulnerable
7	Uning	20,000	College undergraduate	Working full-time (water station manager), formerly an OFW	Vocational	15	Seafarer (Worldwide)	Lower-Middle—Vulnerable
8	Severina	20,000	Elementary	Stay-at-home, no work experience	College undergraduate	20	Construction worker (Saudi)	Lower-Middle—Vulnerable
9	Lisa	25,000	High school	Stay-at-home, previously employed (OFW)	High school	16	Go-kart mechanic (Abu Dhabi)	Lower-Middle—Vulnerable
10	Marie	30,000	High school	Stay-at-home, previously employed	High school	11	Driver (Kuwait)	Middle—Vulnerable
11	Marjorie	30,000	High school	Stay-at-home, previously employed	College	5	Chef (Qatar)	Middle—Moderately Stable
12	Arlene	30,000	High school	Stay-at-home, no work experience	College undergraduate	10	Construction worker (Qatar)	Middle—Moderately Stable
13	Leah	30,000	College	Stay-at-home, previously employed	College	24	Seafarer (Worldwide)	Middle—Moderately Stable
14	Helen	35,000	Vocational	Stay-at-home, previously employed	College	12	Seafarer (Worldwide)	Middle—Moderately Stable
15	Grace	40,000	College	Working part-time (online seller)	College	12	Seafarer (Worldwide)	Middle—Moderately Stable
16	Christine	40,000	College	Stay-at-home, previously employed (businesswoman)	Vocational	22	Seafarer (Worldwide)	Middle – Stable
17	Susan	47,000	College	Stay-at-home, no work experience	College undergraduate	14	Fiber optic worker (Saudi)	Middle—Stable
18	Portia	50,000	Vocational	Working full-time (freelancer, WFH)	College	12	Seafarer (Worldwide)	Middle—Stable
19	Alma	50,000	Vocational	Working part-time (hairdresser/manicurist), formerly an OFW	College	18	Chef (Dubai)	Middle—Vulnerable
20	Analyn	50,000	College undergraduate	Stay-at-home, previously employed	College	16	Seafarer (Worldwide)	Middle—Stable

As many of these women are raising adolescent children, they prioritize fostering propriety and discipline, believing that their effectiveness as mothers directly influences their children’s behavior. One mother, for instance, threatened to discontinue her financial support should her children engage in vices, intending to discourage such practices. Khaye shares,

“*I raised them to be well-behaved children. They do not have any vices because I made it clear that if they chose to develop such habits, they would have to support themselves financially rather than rely on me*”.

Portia similarly feels pressured to raise her daughter from a previous relationship according to socially acceptable norms, particularly as they live with her in-laws. She closely guides her daughter to ensure that her conduct aligns with societal expectations for girls and appropriate mothering. Adopting a strict parenting style is her way of ensuring that her daughter grows well, as she frequently corrects her behavior. Portia often tells her daughter to “get along with everyone” and notes that she “no longer comes out of her room”. Through these reminders, Portia seeks to prevent negative judgments from being directed at her daughter. She also expects her to participate in household chores as a way of demonstrating cooperation and respect within the family.

Similarly, Leah repeatedly admonishes her children to the point of annoying them. She explains, “I sometimes scold them because children these days can be quite unruly. When my youngest acts up and my eldest gets irritated, they end up fighting, and I scold them both”. On the other hand, Cherry advises her son not to retaliate when provoked. She explains, “*Sometimes my son gets into trouble at school, but he tells me he does not fight back even if they gang up on him. As a parent, that upsets me, so I always remind him to be patient, stay away, and report it to a teacher or school staff instead of retaliating*”.

Failure to uphold these expectations often leads to social scrutiny, reinforcing a heightened sense of maternal responsibility. Despite Cherry’s persistent advice to her son, she described being judged as a *“bad mother.”* She said, “*I felt deeply embarrassed—the teacher humiliated me. As a teacher yourself, do you think it is ethical to tell a parent, in front of other parents, that “honestly, I do not see any good behavior in your son?*”.

This parenting style is adopted by mothers with younger children who are less mature for their age. They regard this approach as a salient aspect of mothering. Through this, they view themselves as moral gatekeepers, thereby socially reproducing their traditional roles based on the gendered expectations of Filipino motherhood as they navigate their tasks as lone parents.

#### 4.1.2 Mediating father–child relationships in transnational families

Non-migrant mothers in Filipino transnational families navigate patriarchal structures that position fathers as primary breadwinners and ultimate authority figures, even in their physical absence. Economic provision from abroad becomes a key mechanism through which paternal authority is sustained, shaping family decision-making processes. Christine’s husband regularly sends 40,000 pesos (approximately 680 US dollars), which generally covers their household expenses. Larger financial obligations such as housing amortization for their children’s condominium, investments, insurance, and major educational expenses are independently shouldered by her husband. Through this stable economic support, his overseas employment secures financial stability and reinforces his position as the primary decision-maker within the household. This arrangement requires financial transparency on Christine’s part, as she regularly accounts for household expenditures and consults her husband on major purchases. In this way, financial provision functions not only as economic support but also as a mechanism through which mediated patriarchy is maintained. According to Christine,

“*My husband is kind, though not without flaws. Disagreements occasionally arise, particularly around household spending. He expects financial transparency and requires that expenses be explained, as he closely monitors how money is allocated. Within marriage, decision-making authority is ultimately vested in the husband. Although the wife may offer suggestions, final decisions align with his judgment. This is consistent with biblical principles of marital submission*”.

Lisa similarly reproduces mediated patriarchy. Although she has access to her husband’s ATM and online banking, she does not exercise autonomy over their finances. She is required to consult her husband regarding major household expenses, reinforcing his authority over financial decision-making.

However, mediated patriarchy in transnational families is not confined to economic provision alone; it is also reproduced through everyday practices that sustain paternal authority across distance. Beyond financial support, mothers actively facilitate regular communication between their OFW husbands and children, enabling the continued symbolic presence of fathers as well as forms of surveillance and parenting from afar.

Within this arrangement, mothers are expected not only to manage household and child-rearing responsibilities but also to actively uphold their husbands’ stature within the family. Beyond these responsibilities, non-migrant mothers actively mediate the relationships between their children and their overseas fathers. They involve their husbands in decision-making regarding their children. According to Rosie,

“*We have internet access, and my husband regularly checks on the children. When they ask permission to go out, I inform them that I cannot allow it without their father’s approval, which they can obtain online”. Younger mothers emphasize the need to foster close father–child bonds despite geographical separation. One informant shared, “Even though their dad is far away, I want them to feel that he is close, someone they can talk to about anything. In everything they do, you should be supportive, so they truly feel it*”.

This dynamic also involves encouraging OFW husbands to actively co-manage the parenting of their adolescent children. Majorie explained, “*They are monitored by their father. For example, I inform him of their stubborn attitude, which is why I scold them*”. Myra noted that she regularly reports her children’s misbehavior to her husband and observed that they are more likely to comply when both parents jointly enforce discipline. These accounts suggest that strengthening parent–child relationships requires fathers to maintain a sense of authority and involvement, even from afar.

This effort highlights the dual emotional labor of non-migrant mothers, who not only provide direct care for their children but also ensure that the absent father remains an influential figure in their lives. By maintaining these ties, non-migrant mothers work to preserve family cohesion despite the spatial and emotional distance caused by migration.

However, mediated patriarchy is actively negotiated by some of the informants. Khaye shared several experiences that illustrate how she challenged patriarchal expectations within her family. When she and her husband were newlyweds, they initially lived in Manila. Later, her in-laws requested that they move to the province and live with them. Khaye asserted her conditions, explaining, “*Your son has his own family now. We will only live in the province if we have a separate house. Your son is already a family man and should stand on his own feet rather than rely on your support*”. Through this response, Khaye affirmed her husband’s responsibility as a provider but also demonstrated that she exercised authority within the marriage. In addition, she encouraged her husband to work abroad with the aim of improving their family’s financial situation. However, due to irregular and insufficient remittances he sent, she rejected financial dependence and challenged her husband’s opposition to her seeking paid work. The demands of motherhood compelled her to assume the role of breadwinner to ensure her children’s economic security. In doing so, she not only fulfilled maternal responsibilities but also actively negotiated and challenged mediated patriarchy.

#### 4.1.3 Financial adaptation and the economic role of non-migrant mothers

While caregiving remains central to their responsibilities, financial adaptation emerges as another critical aspect of sustaining family stability. In most transnational families, remittances are directed to non-migrant mothers, making them the primary financial managers. However, irregular and often insufficient remittances present significant challenges.

Many OFW husbands in the study are employed in construction-related jobs, automotive services, and other low-wage sectors in the Middle East. Over half of the informants reported receiving remittances ranging from 10,000 to 30,000 pesos (approximately 250–500 US dollars), often sent irregularly. This uncertainty forces non-migrant mothers to seek alternative income sources, particularly in the informal sector.

These include seasonal farm labor (Cherry), long-term domestic work (Khaye), overseas and home-based services such as pedicure, manicure, and hair treatment (Alma), transitions from formal employment to flexible online work (Grace), online or home-based work (Portia), involvement in family enterprises (Uning), and permanent government employment (Carol).

This pattern underscores that even within transnational families, the unreliability and insufficiency of remittances necessitate maternal labor as a substitute for unstable income flows. These mothers are resourceful and find ways to address financial deficits when their husbands fail to send remittances. Their capacity to act as co-providers ranges from survival-level to strong, reflecting differences in socio-economic positioning. However, mothers from low socio-economic backgrounds have a nuanced understanding of their role as providers.

For instance, Khaye, who worked as a house helper for 11 years, may contribute less to the family’s financial resources compared to her husband, who sends around 14,000 pesos (approximately 250 US dollars), but she considers herself the family’s breadwinner, given her efforts to meet the family’s financial needs. This includes borrowing money from her siblings or her employer. According to Khaye,

“*Many people have asked why I do not oblige my spouse to send money. I am the one who figures everything out because when my spouse says there is nothing, there is nothing I can do. Even if I argue, it will not make a difference”*.

On the other hand, Cherry reports working as a seasonal farm worker—specifically in onion planting and harvesting—when household finances are insufficient. Her husband’s remittances of 16,000 to 21,000 pesos (272–357 US dollars) per month are often inadequate to meet their children’s needs. With limited support, as both her parents are deceased, her two eldest sons have also engaged in occasional farm work to save money for their necessities. Her participation in the labor force is survival-driven and serves as a supplemental means to augment the family’s finances. She said, “*Sometimes remittances are not enough because the prices of goods keep on rising and I have four children. During the onion planting and harvesting seasons, I work as a laborer when needed, usually about thrice a week”*. Unlike Khaye, she does not consider herself the family’s breadwinner.

In contrast, Portia and Grace—both wives of seafarers—occupy a relatively different socio-economic position. Although the remittances they receive are higher than those of other informants in this study, they reported experiencing pressure to help support their families due to their husbands’ employment interruptions, often caused by illness or the inability to immediately return to work. Unlike other OFWs who sign their contracts before returning to the Philippines for vacation, seafarers must wait to be queued for their next deployment, illustrating the precarity of their work.

The situation is different for Carol—a midwifery graduate with 17 years of professional experience in both private and public hospitals in the country. Although her husband works as an automotive mechanic in Saudi Arabia, she occupies a central breadwinning role in the family and asserts that even without overseas remittances, she can provide for her children since her salary is higher than her husband’s remittances. In fact, she does not obligate her husband to send money regularly, emphasizing instead that he should prioritize saving abroad, reflecting her financial independence and confidence in her ability to sustain the household if necessary.

Collectively, these narratives challenge the assumption that overseas labor migration uniformly guarantees household financial security through remittances. Instead, they reveal that the responsibility of ensuring economic stability often continues to rest on non-migrant mothers, albeit in ways that vary according to their access to education, employment opportunities, income stability, and support systems. Through their economic resourcefulness and co-provider roles, working non-migrant mothers not only ensure the financial stability of their families but also maintain social cohesion in marital and parent–child relationships.

This endeavor reveals inherent contradictions. Non-migrant working mothers experience ambivalence as they strive to maintain traditional gender roles while their husbands work overseas, even as they assume breadwinning responsibilities. This is consistent with the findings that among Filipino working mothers, the experience of maternal ambivalence is connected to their insufficient time to spend with their children owing to their dedication to work duties (Liwag et al., 1998). Although these mothers contribute financially to their families, they continue to prioritize their mothering roles—such as nurturance, caregiving, and managing household responsibilities—over paid work. This is reflected in Cherry’s experience: “*At times, farm work offers overlap with the schedule for submitting my children’s modules, so I am unable to accept the work and must prioritize their submission*”. This illustrates how, within transnational families, the welfare of children remains paramount despite women’s increasing economic participation.

Working non-migrant mothers employ two main strategies to resolve the internal contradictions brought about by their employment. Some mothers choose to give up paid work to focus on raising their children. Although Rosie earned a college degree, she eventually gave up work. According to Rosie,

“*When the idea of going abroad came up, I thought about what would happen to my children if I left them behind, especially since we were living with my in-laws at the time. Later, my husband said that he would be the one to work overseas instead. He was determined to do it and believed that it was better for a woman to take care of the children. That became my reason. I did not want to leave them. I wanted to be the one to look after them*”.

On the other hand, Christine felt pressured by her children to choose them over managing their pharmacy. According to her,

“When I arrive, of course it is already nighttime, and when I leave, they are still asleep. That is how it is. Before, the only thing they would say to me was, “You are always at the pharmacy!” *They said that I was busy with business, because they did not understand yet. They did not know that there was money in it to support them. They felt like I was neglecting them. That was how they felt back then”*.

Others, however, persist in seeking employment, either by negotiating with or defying their husbands’ preferences. These mothers ensure they can balance their time between work and childcare.

For instance, mothers like Portia and Grace opted to work online, while Alma worked as a manicurist, pedicurist, and hairstylist. Portia and Grace received support from their immediate family or their husbands’ kin while engaged in online work. Grace reflected on her experience working at a casino, highlighting the sacrifices she made to fulfill both work and family obligations. She traveled approximately 4 h from Manila to attend her son’s school program and then returned immediately for work, demonstrating her efforts to maintain a work–life balance. Similarly, when Christine was managing their pharmacy, she shared that before leaving home, she made sure her children’s meals were already prepared.

### 4.2 Communication dynamics and emotional support in Filipino OFW families

Based on the profiles of informants’ OFW husbands, 14 are working in land-based jobs, mostly in various industries in the Middle East, while six are working as seafarers. In terms of the length of time working as OFWs, their husbands have been employed for a minimum of two and a maximum of 24 years, averaging 12.3 years in their migration stint. These long years of overseas work are intended to provide financial stability for non-migrant families. This goal has prompted some OFW husbands to delay their vacations in the Philippines and instead request their employers to convert their travel allowances into cash so they can send it to their respective families for household use. This situation illustrates the trade-off of prioritizing the welfare of their family over quality time together and physical proximity.

The impact of OFW migration transcends economic provision and physical separation. It is accompanied by the interactional and emotional adjustments needed to maintain familyhood in the process. To maintain close relationships with their non-migrant children, open communication is facilitated by non-migrant mothers between OFW husbands and their children, emphasizing fathers’ breadwinning roles over physical family reunifications. This underscores the significant role non-migrant mothers play in the adaptation and functioning of OFW families.

Given that the informants in this study are mothers of adolescent children, their emotional resilience—maintained through transnational communication—is important in sustaining connections between OFW fathers and their children. These mothers actively reinforce the image of their OFW husbands as dedicated providers who sacrifice physical presence for the family’s welfare. Through consistent communication via social media, they help bridge the emotional and physical distance among family members. The active involvement of non-migrant mothers is therefore crucial in preserving family cohesion and a sense of “familyhood,” despite the prolonged absence of the primary breadwinner.

Christine shared that in the past, there were no cellphones through which she could communicate with her seafaring husband. Her husband initiated calls only when their ship was docked and telecommunication services were available. As a dutiful wife, she patiently waited for her husband’s overseas call. Times have changed, and it is now possible for them to communicate three times a day. This change illustrates the positive impact of technology and how OFW families leverage it to transcend the challenges accompanying overseas labor migration among Filipinos.

Despite extended physical separation, some families report stronger emotional connections due to consistent communication. Helen observed, “We became even closer. Just like before, because almost every day during his break time, he would call. He has not been home for 6 years now*”*. These accounts reveal how transnational communication serves as an emotional bridge, enabling the family system to maintain its interconnectedness and balance even in the face of prolonged absence.

Non-migrant mothers play a pivotal role in maintaining and shaping family dynamics within transnational families, particularly through their active engagement in transnational communication. Acting as a vital bridge, they foster connections between their adolescent children and their OFW husbands, enabling a sense of cohesion and continuity despite physical separation. Through regular and intentional communication, non-migrant mothers facilitate the practice of “*doing family*” across borders. This dual-faceted process strengthens the emotional bond between OFW fathers and their adolescent children while simultaneously reinforcing maternal authority. By involving OFW husbands in critical parenting decisions and discussions, non-migrant mothers effectively mitigate tensions that might arise in their relationships with their children. This collaborative approach not only enhances family stability but also underscores the intricate interplay of roles and relationships within transnational families.

For instance, when their adolescent children need emotional support, non-migrant mothers involve their OFW husbands. Grace shared that her 13-year-old son struggled with obsessive-compulsive disorder and anxiety amid the pandemic. She observed his persistent hand and foot washing as a precaution against the coronavirus. To demonstrate their support, both parents engaged in conversations with their son and provided reassurance of their unwavering presence. She said,

“*Every day, we try to communicate with him. We encouraged him to express his feelings. I reassured him to open, and my spouse did the same. Over time, the emotions that once felt overwhelming gradually subsided. We always start by talking—he shares his feelings with us, although it is not always easy for him*”.

However, transnational communication, despite its significant role in sustaining familyhood among transnational families, also has notable drawbacks. First, it can reinforce mediated patriarchy, particularly in households where non-migrant mothers are full-time caregivers. Through constant communication, migrant fathers continue to assert authority over childrearing and discipline, thereby limiting mothers’ autonomy in these roles.

Second, transnational communication enables forms of surveillance, allowing OFW husbands to monitor their non-migrant wives’ daily activities, caregiving practices, and management of remittances from a distance. Analyn shares that although she and her husband live separately, his constant phone calls make it feel as though he is still physically present in the household. According to her,

“*He is very jealous. Before, my mother-in-law would often take me along wherever she went, and he would get angry about that. He would ask where we were going and accuse us of going places unnecessarily. Even when I just ride a tricycle, he wants me to take different ones each time, as if he is jealous of the drivers. He has tendencies like that, so I always bring my child with me*”.

Regular communication becomes a means of assessing whether children are being properly raised and disciplined. As Leah explains, “You really need to have a lot of patience. You always must keep an eye on them because their dad wants them to be well taken care of and well-behaved”. This illustrates how paternal expectations are continuously enforced despite physical absence. Oftentimes, major household expenses are discussed with husbands, whose approval is required before any decision is made. All quotations have been translated by the author.

## 5 Discussion

This paper discusses the role played by non-migrant mothers in OFW families. The migration of OFW husbands heightens non-migrant mothers’ sense of responsibility for both the emotional wellbeing and moral education of their children. This finding is supported by Aryal et al. (2020), who examined the departure of men and the ensuing psychosocial stress encountered by wives left behind in Nepal. Nguyen et al. (2006), in their analysis of migration trends in Asia, noted that such movements have resulted in heightened responsibilities, increased burdens, and financial struggles for the wives left behind.

Sociological analyses highlight parenthood as a gendered institution, predominantly assigning childrearing responsibilities to mothers rather than fathers (Brown, 2011; Madhavan et al., 2008). These notions encompass the societal ideals placed upon mothers, aligning with both the essentialist view of nurturing and childcare (Barlow and Chapin, 2010) and broader roles such as being moral gatekeepers (Liamputtong, 2006). This ideology perpetuates societal expectations (Ross, 2016), significantly influencing women’s behaviors and shaping their role expectations (Hays, 1996).

Filipino non-migrant mothers’ adherence to traditional expectations of motherhood serves as a stabilizing force in transnational family dynamics. Their commitment to childcare and household duties ensures that their children experience continuity in care and emotional support, which are critical components of family cohesion. By stepping into this intensified caregiving role, non-migrant mothers bridge the gap left by the physical absence of OFW fathers, fostering a sense of security and normalcy within the household. Additionally, the reinforcement of the intensive motherhood ideology reflects an adaptive strategy to uphold familial bonds and relational stability. Through their efforts, non-migrant mothers not only meet the practical needs of their children but also cultivate an environment where familial values, routines, and emotional connections are maintained. This active participation strengthens the relational fabric of the family, ensuring that the family unit remains cohesive despite the challenges posed by geographical separation.

In this context, the traditional expectations of motherhood become instrumental in maintaining family cohesion, as they enable non-migrant mothers to act as anchors who preserve the structure and emotional wellbeing of the family amidst the pressures of overseas labor migration.

The study also revealed the short-term economic viability of overseas work, as OFWs are often compelled to engage in cyclical migration to sustain their families (Manapol et al., 2022). Anday et al. (2024) found that OFW fathers primarily view their role as financial providers, ensuring their family’s stability and securing a bright future for their children. Additionally, they see themselves as disciplinarians, responsible for correcting their children’s misbehavior. This perspective is rooted in the belief that their children’s conduct outside the home reflects their effectiveness as parents. Given the societal expectation for OFW husbands to provide better financially, non-migrant mothers often feel compelled to ensure their children’s adherence to discipline and academic success. This view aligns with the broader cultural belief in the Philippines that fatherhood is a legacy handed down through generations (Alberto, 2015).

Fathering is gendered. According to this perspective, the father is responsible for imparting his values and beliefs about life, masculinity, and, ideally, fatherhood to his son, shaping him into a reflection of his ideals. Marquez (2022) discovered a prevailing trend among migrant Cocas fathers with young children in the United States and Mexico: they were determined to maintain their authority before migrating. Despite being physically distant, these fathers remained vocal, opinionated, and engaged in their children’s upbringing. In contrast to previous generations, they saw themselves as significantly more involved and less emotionally detached fathers.

Filipino non-migrant mothers play a vital role in helping their OFW husbands maintain their roles as fathers to their non-migrant children. This has led to a greater involvement of migrant husbands in the day-to-day lives of their non-migrant families, as observed in the literature. Jahan (2021) contended that the migrant husband’s involvement in the lives of other transnational family members constitutes a form of mediated patriarchy, perpetuating gender surveillance. Madianou (2016) reframes this situation using the concept of “*ambient co-presence,”* which denotes the heightened awareness of separated family members through the pervasive presence of social media. This concept holds particular significance for transnational families that rely on communication technologies to sustain long-distance relationships. Filipino non-migrant mothers consider regular communication with their OFW husbands crucial for strengthening their relationships, not only in the marital context but also in the father–child dynamic.

Filipino non-migrant mothers’ roles go beyond facilitating the collaborative parenting of their OFW husbands through transnational communication. Some of them co-provide alongside their OFW husbands. However, they must maintain the image of a “good mother” in the eyes of their respective families. Thus, they must balance their professional responsibilities with familial duties, prioritizing involvement in their children’s school-related activities over work engagements. In striving for this balance, non-migrant mothers aim to mitigate any guilt stemming from their divided attention between work and family, ultimately seeking to safeguard their children’s wellbeing while maintaining their financial independence and professional pursuits.

### 5.1 Implication for research

Future research may build on the findings of this study by examining transnational families across different temporal and contextual dimensions. Longitudinal studies that follow families through various stages of migration—pre-departure, during migration, and post-return—would provide deeper insights into how family cohesion, roles, and relationships evolve over time.

In addition, comparative studies across different regions (e.g., the Middle East, East Asia, and Western countries) could illuminate how varying labor conditions, migration policies, and cultural contexts shape family dynamics. To complement qualitative approaches, quantitative studies may also be conducted to measure levels of family cohesion and wellbeing among OFW and non-OFW families using standardized scales.

To ensure a more inclusive understanding of transnational family life, future studies should place greater emphasis on the lived experiences of non-migrant family members, including spouses, children, and elderly parents who remain in the Philippines. Qualitative methodologies such as narrative inquiry or phenomenological analysis may be particularly useful in capturing how these individuals cope with prolonged separation, manage emotional challenges, and sustain family bonds across distances.

Since the current paper explored factors promoting family cohesion, a logical next step is to investigate the complex dynamics of family disintegration and marital dissolution within the context of transnational migration. Future studies should identify socio-economic and psychological stressors that contribute to these issues. The findings from such research could be used to develop evidence-based and culturally sensitive interventions and counseling programs tailored to the unique challenges of migrant families. These programs could be offered by government agencies, non-governmental organizations, or counseling centers to help families develop strategies for managing conflict, improving communication, and strengthening their relationships.

In terms of practical implications, the findings of this study highlight the need to enhance pre-departure orientation programs for OFWs to better prepare both migrants and their families for the realities of transnational family life. Furthermore, government agencies and non-governmental organizations should support the establishment of community-based support structures for non-migrant mothers, including financial literacy training, livelihood and entrepreneurship programs, and accessible mental health services. Such initiatives may help mitigate the emotional and economic burdens associated with prolonged spousal absence and contribute to the overall wellbeing and resilience of transnational families.

### 5.2 Limitations

The locale of the study is a rice-producing province, approximately 100 miles from Manila, the capital of the Philippines, accessible by bus or private car. The socio-economic context in the province may differ from that of other regions. Thus, regional variation based on poverty indices may influence the transferability of findings. A region with a higher incidence of poverty might show greater involvement of non-migrant mothers in paid employment to ensure family survival, whereas regions that strongly embrace the traditional nurturing role of mothers, consistent with the intensive motherhood ideology, higher fertility rates, and larger family sizes with young children, might discourage mothers from co-providing with their OFW husbands.

The findings may also be less applicable to higher-income groups. Additionally, researcher positionality—being both a local teacher and mother—adds contextual depth but necessitates reflexivity to minimize bias. Reflexive practices, such as journaling and peer debriefing, were employed to ensure objectivity, though the study’s context-bound nature limits broader generalizability (Mayring, 2007).

The use of referral-based sampling may also incur potential selection bias. In addition, the absence of triangulation with children’s and fathers’ perspectives might affect the interpretation of family dynamics. This study illustrates only mothers’ perspectives and narratives. Moreover, maternal mediation perceived as promoting family cohesion may be interpreted differently by their children.

While remote qualitative data collection methods, such as conducting online interviews during the pandemic, are both relevant and practical, they also present limitations. Datoon et al. (2022) highlight the challenge of building rapport with respondents during phone interviews. Unlike face-to-face interviews, which may foster rapport and encourage informants to share more of their lived experiences, remote methods may not facilitate the same level of connection. However, because the informants in this study were referred by a former student, friend, or relative, trust and comfort were more readily established.

## 6 Conclusion

This study highlights the multifaceted and critical role of non-migrant mothers in maintaining cohesion in overseas Filipino families. Their narratives reveal that while the overseas labor migration of their husbands ensures the financial security of their families, the responsibility of maintaining family stability rests on the shoulders of non-migrant mothers. The findings highlight two major themes: the instrumental role of non-migrant mothers in sustaining family cohesion and the pivotal role of communication dynamics and emotional support. Economically, the study challenges the notion of remittance as a stable source of financial security, revealing that irregular and insufficient remittances compel non-migrant mothers to become economic co-providers. This illustrates that non-migrant mothers are not merely passive recipients of remittances but are proactive agents of family resilience. This study concludes that recognizing and supporting the roles of non-migrant mothers is paramount for the long-term wellbeing of OFW families.

## Data Availability

The raw data supporting the conclusions of this article will be made available by the authors, without undue reservation.
